# STIM Protein-NMDA2 Receptor Interaction Decreases NMDA-Dependent Calcium Levels in Cortical Neurons

**DOI:** 10.3390/cells9010160

**Published:** 2020-01-09

**Authors:** Joanna Gruszczynska-Biegala, Klaudia Strucinska, Filip Maciag, Lukasz Majewski, Maria Sladowska, Jacek Kuznicki

**Affiliations:** 1 Laboratory of Neurodegeneration, International Institute of Molecular and Cell Biology in Warsaw, 02-109 Warsaw, Poland; klaudiastrucinska@gmail.com (K.S.); fmaciag@iimcb.gov.pl (F.M.); lmajewski@iimcb.gov.pl (L.M.); m.sladowska@cent.uw.edu.pl (M.S.); jacek.kuznicki@iimcb.gov.pl (J.K.); 2 Molecular Biology Unit, Mossakowski Medical Research Centre Polish Academy of Sciences, 02-106 Warsaw, Poland

**Keywords:** STIM proteins, NMDA receptor, neuronal store-operated calcium entry (nSOCE), endoplasmic reticulum (ER), plasma membrane (PM), neurons, organellar Ca^2+^, Ca^2+^ homeostasis

## Abstract

Neuronal Store-Operated Ca^2+^ Entry (nSOCE) plays an essential role in refilling endoplasmic reticulum Ca^2+^ stores and is critical for Ca^2+^-dependent neuronal processes. SOCE sensors, STIM1 and STIM2, can activate Orai, TRP channels and AMPA receptors, and inhibit voltage-gated channels in the plasma membrane. However, the link between STIM, SOCE, and NMDA receptors, another key cellular entry point for Ca^2+^ contributing to synaptic plasticity and excitotoxicity, remains unclear. Using Ca^2+^ imaging, we demonstrated that thapsigargin-induced nSOCE was inhibited in rat cortical neurons following NMDAR inhibitors. Blocking nSOCE by its inhibitor SKF96365 enhanced NMDA-driven [Ca^2+^]_i_. Modulating STIM protein level through overexpression or shRNA inhibited or activated NMDA-evoked [Ca^2+^]_i_, respectively. Using proximity ligation assays, immunofluorescence, and co-immunoprecipitation methods, we discovered that thapsigargin-dependent effects required interactions between STIMs and the NMDAR2 subunits. Since STIMs modulate NMDAR-mediated Ca^2+^ levels, we propose targeting this mechanism as a novel therapeutic strategy against neuropathological conditions that feature NMDA-induced Ca^2+^ overload as a diagnostic criterion.

## 1. Introduction

As a critical intracellular signaling ion, calcium (Ca^2+^) coordinates numerous cellular processes, such as fertilization, proliferation, development, learning, and memory [1]. The main store of intracellular Ca^2+^ ions is the endoplasmic reticulum (ER). Maintaining intracellular Ca^2+^ homeostasis is vital for cell survival [2], which underscores the need to elucidate the mechanisms underlying cellular Ca^2+^ dynamics. In non-excitable cells, such as lymphocytes, extracellular Ca^2+^ influx through the tightly regulated store-operated Ca^2+^ channels (SOCCs) in the plasma membrane (PM) drives store-operated Ca^2+^ entry (SOCE) [3] that regulate Ca^2+^ influx. SOCE is activated after sensing Ca^2+^ levels in the ER through a mechanism using the Stromal Interacting Molecules (STIM) proteins, STIM1 and STIM2 [4,5,6]. STIM proteins were initially discovered as potential tumor suppressor proteins [7]. Emptying ER Ca^2+^ stores induces oligomerization of STIM proteins and migration of the oligomers towards ER regions juxtaposed to the PM [4,6,8], where they form complexes with Ca^2+^-selective channels like Orai1/2/3 [4,9,10,11,12] and/or non-selective channels from the Transient Receptor Potential (TRP) family [13,14]. These complexes are termed “puncta”. So, Ca^2+^ flows into the cytoplasm [15] and then into the ER by activating the sarco/-endoplasmic reticulum Ca^2+^ ATP-ase (SERCA) pump to refill this intracellular store with Ca^2+^ [3,16].

However, our knowledge on neuronal SOCE (nSOCE) remains limited. In fact, the role of STIM proteins in this process is debated given the complex regulation of Ca^2+^ inflow through various receptors and Ca^2+^ channels [17,18,19]. nSOCE was identified in cortical, hippocampal, cerebellar, dorsal root ganglion, and dorsal horn neurons (reviewed in [19,20,21]) and shown to regulate synaptic plasticity, neurotransmitter release, and gene expression [22,23,24,25,26]. The complex relationships among various Ca^2+^ influx pathways in neurons have only been partly elucidated. When STIM1 is activated by neuronal store depletion, it not only activates SOCCs, but also inhibits voltage-gated Ca^2+^ channels (VGCCs) [27,28,29]. In addition, STIM proteins contribute to metabotropic glutamate receptor 1 (mGluR1)-dependent synaptic transmission [25] and modulate α-amino-3-hydroxy-5-methyl-4-isoxazolepropionic acid receptor (AMPAR) activity by interacting with its subunits [30,31]. In pyramidal neurons, SOCE, possibly activated by *N*-methyl-d-aspartate receptor (NMDAR) stimulation, may contribute to synaptic plasticity [32]. 

However, Ca^2+^ entry in neurons primarily remains under control by well-defined (VGCCs), which are localized in the cell soma, dendrites, and nerve terminals [33], and receptor-operated channels (ROCs), such as AMPARs and NMDARs, that function at both synaptic and extrasynaptic sites [34,35,36]. NMDARs belong to the family of ionotropic receptors that respond to extracellular glutamate. They contribute to key cellular processes like synaptic development, neuronal excitability, synaptic plasticity and learning and memory [37,38,39]. NMDAR-mediated Ca^2+^ influx provides a significant source of Ca^2+^ entry through PM channels, and upregulation of NMDAR activity was implicated in excitotoxicity and cell death associated with neurological disorders, such as traumatic brain injury, ischemia, Alzheimer’s disease (AD), Huntington disease (HD), schizophrenia, and mental impairment [40,41,42,43]. 

We earlier showed that STIM-dependent nSOCE operates in cultured cortical neurons [31,44,45]. However, a potential role of STIM proteins in NMDA-induced Ca^2+^ influx remains unreported, to our knowledge. We hypothesized that STIM proteins directly interact with NMDARs to modulate neuronal activity. Here, we address this issue in vitro and in situ in cultured cortical neurons. We show that nSOCE is attenuated by NMDAR antagonists and NMDA-induced intracellular Ca^2+^ level depends on STIM proteins. In addition we demonstrate a physical interaction between NMDAR subunits and STIM proteins by proximity ligation assay, immunocytochemistry, and co-immunoprecipitation. Together, our findings reveal that STIMs are potential negative regulators of NMDA-stimulated Ca^2+^ signaling in cortical neurons. 

## 2. Materials and Methods

### 2.1. Experimental Design

Please see the Table 1 for the overall experimental design for this work.

### 2.2. Primary Cell Cultures

Cortical neuronal cultures were prepared from embryonic day 19 (E19) Wistar rat brains or wild type (FVB/NJ; WT) and transgenic Tg(STIM1)Ibd mouse brains as previously shown [23,45]. Pregnant female Wistar rats were provided by the Animal House of the Mossakowski Medical Research Centre, Polish Academy of Sciences (Warsaw, Poland) and WT/Tg mice were provided by the Animal House of the Nencki Institute of Experimental Biology, Warsaw, Poland. Animal care was in accordance with the European Communities Council Directive (86/609/EEC). The experimental procedures were approved by the Local Commission for the Ethics of Animal Experimentation no. 1 in Warsaw (656/2015 and 416/2017). Brains were removed from rat embryos and collected in cold Hanks solution supplemented with 15 mM HEPES buffer and penicillin/streptomycin. The cortices were isolated, rinsed three times in cold Hanks solution, and treated with trypsin for 35 min. The tissue was then rinsed in warm Hanks solution and dissociated by pipetting. For the Co-IP assays and WB, neurons were seeded on poly-D-lysine-precoated BioCoat plastic Petri dishes (Corning, Tewksbury, MA, USA) at a density of 7 × 10^6^ cells/plate. For Ca^2+^ measurements, primary cortical neurons were plated at a density of 7 × 10^4^ cells/well on eight-well PDL-laminin-precoated chamber slides (BioCoat, Bedford, MA, USA). For IF and PLA measurements, neurons were plated at a density of either 17 × 10^4^ per 13 mm glass coverslip or 4 × 10^4^ per glass well in 16-well chamber slides (Lab-Tek, Nunc, Rochester, NY, USA) coated with laminin (2 µg/mL; Roche, Mannheim, Germany) and poly-D-lysine (38 µg/mL; Sigma-Aldrich, St. Louis, MO, USA), respectively. Neurons were grown in Neurobasal medium (Gibco, Paisley, UK) supplemented with 2% B27 (Gibco), 0.5 mM glutamine (Sigma), 12.5 μM glutamate (Sigma), and a penicillin (100 U/mL)/streptomycin (100 mg/mL) mixture (Gibco). Cultures were maintained at 37 °C in a humidified 5% CO_2_/95% air atmosphere. Every 3–4 days, half of the conditioned medium was removed and replaced by fresh growth medium. The experiments were performed on 15-day-old cultures (Co-IP assay) or 16- to 17-day-old cultures (Ca^2+^ measurements, WB, IF, and PLA).

### 2.3. Cell Line Culture

HeLa cells or HEK 293T/17 cells (provided from ATTC) were grown in Dulbecco’s modified Eagle’s medium (DMEM) that contained 10% fetal bovine serum and a penicillin (100 U/mL)/streptomycin (100 mg/mL) mixture (Gibco) at 37 °C in a 5% CO_2_ atmosphere.

### 2.4. Transfections

YFP-STIM1 and YFP-STIM2 constructs were a generous gift from Dr. Tobias Meyer, Stanford University. Cortical neurons grown on eight-well PDL-laminin-precoated chamber slides were transiently transfected with the aid of Lipofectamine 2000 Reagent (1 µL per well; Invitrogen, Carlsbad, CA, USA) according to the manufacturer’s protocol. YFP-STIM constructs (STIM1 or STIM2) were used at 0.35 µg of DNA per well. As a control, 0.35 µg of YFP cDNA per well was used. The cells were exposed to the mixture of plasmid DNA and Lipofectamine 2000 for 1 h in a serum-free culture medium. Afterward, the neurons were returned to saved conditioned Neurobasal supplemented with 2% B27, 0.5 mM glutamine, 12.5 µM glutamate, and penicillin/streptomycin. The experimental treatments were initiated 24 h after transfection.

### 2.5. Virus Production and Transduction

For STIM1 and STIM2 silencing, commercially available 4 short-hairpin RNA (shRNA) sequences in pLenti-GFP (Origene, Rockville, MD, USA) were used (A, B, C, D). shRNA B turned out to be toxic to neurons, which is why it was not used in further experiments. As a control, scrambled shRNA against pLenti-GFP was applied (sc) (Origene). The knockdown of STIM1 or STIM2 was performed by transducing the cells with lentiviruses that carried pLenti-GFP plasmids that targeted shRNAs against the STIM1 or STIM2 sequence or scrambled shRNA as a control. The viruses were prepared in HEK 293T/17 cells by the Ca^2+^ phosphate transfection method. Seventy-two hours after transfection supernatants were collected, filtered through 0.45 μm membranes, concentrated in Vivaspin 100-kDa units (Sartorius) in a swing-out rotor at 1000× *g*, aliquoted and stored at −80 °C until needed. Day in vitro 4 (DIV4) neurons were transduced with lentiviruses to silence STIM1 or STIM2. For viral infection, efficiency was ~40%. Experiments started 12 days after virus transduction.

### 2.6. Electrophysiology

#### 2.6.1. Slice Preparation

All chemicals were purchased from Sigma unless indicated otherwise. Brain sections were prepared using the recently described protective recovery method [46]. After sacrifice of 4-week-old mice by cervical dislocation, the brains were removed and submerged in ice-cold *N*-methyl-d-glucamine (NMDG)-based solution that contained, in mM: NMDG, 92, KCl, 2.5, 4-(2-hydroxyethyl)-1-piperazineethanesulfonic acid (HEPES), 20, thiourea, 2, glucose, 25, NaH_2_PO_4_, 1.25, NaHCO_3_, 30, MgSO_4_, 10, CaCl_2_, 0.5, Na-ascorbate, 5, Na-pyruvate, 3 and 12 mM *N*-acetyl-l-cystein (NAC, myprotein.com). The cerebellum was removed by trimming the brain along the coronal plane. The anterior part of the brain was glued onto the cutting stage with the cut plane bottom and the ventral part facing the blade. Next, 350 μm thick coronal slices were cut with a vibratome (HM 650 V, Thermo Scientific) in NMDG-based solution constantly bubbled with carbogen (95%/5% CO_2_/O_2_). The slices were next transferred to a chamber filled with carbogenated NMDG-based solution (solution temperature was 32 °C). The incubation time and timing of the NaCl solution addition were carried out according to [46]. Following initial recovery, the sections were transferred to an incubation chamber filled with carbogenated HEPES-artificial cerebrospinal fluid (HEPES-aCSF) that contained, in mM: NaCl, 82, KCl, 2.5, HEPES, 20, thiourea, 2, glucose, 25, NaH_2_PO_4_, 1.25, NaHCO_3_, 30, MgSO_4_, 2, CaCl_2_, 2, Na-ascorbate, 5, Na-pyruvate, 3 and 12 mM NAC, and heated to 25 °C. The sections were incubated for 1 h before the recordings. The pH of all solutions was adjusted to 7.3–7.4 at room temperature with carbogen and HCl (NMDG-based solution) or carbogen and NaOH (HEPES-aCSF). The osmolarity was 310 ± 10 mOsm/kg H_2_O. The recording solution (aCSF) was free of Mg^2+^ ions and contained, in mM: NaCl, 126, KCl, 2.6, glucose, 20, NaH_2_PO_4_, 1.25, NaHCO_3_, 25 and CaCl_2_, 2.5, supplemented with 100 μM glycine. The pH of the aCSF was adjusted to 7.3–7.4 at room temperature with carbogen and HCl, and the osmolarity was 315 ± 5 mOsm/kg H_2_O. Throughout the recordings, the extracellular solution was carbogenated and heated with an in-line heater (catalog no. 64-0102, controlled by a TC-324B temperature control unit, Warner Instruments, Hamden, CT, USA) to maintain the temperature close to 32 °C. The rate of solution flow was 4–5 mL/min.

#### 2.6.2. Patch-Clamp Recordings

Pipettes were prepared with the use of a horizontal puller (P-1000, Sutter Instruments, Novato, CA, USA) from borosilicate glass (Warner Instruments, 0.86 mm inner diameter, 1.50 mm outer diameter). Pipette tip electrical resistance was between 2 and 5 MΩ (when filled with internal solution containing, in mM: K-gluconate, 116, KCl, 6, NaCl, 2, HEPES, 20, EGTA, 0.5, adenosine triphosphate-Mg, 4, guanosine triphosphate-Na, 0.3 and Na_2_-phosphocreatine, 10. The osmolarity was adjusted to 300 ± 5 mOsm/kg H_2_O. Currents were recorded from pyramidal cells in the layer V of the cortex in a standard voltage-clamp whole-cell mode. The holding potential was equal to −60 mV. The liquid junction potential was not corrected for (it and was equal to 15.2 mV as calculated in Clampex). Access resistance was monitored every 50 s with a 100 ms long, −5 mV pulse and was electronically compensated in case it exceeded 25 MΩ. Currents were evoked by the application of extracellular solution (aCSF) that was supplemented with 100 μM Na-glutamate. The solution was applied by an automated perfusion system (ValveLink 8.2, Science Products) using Perfusion Pencil (Science Products) with a 100 μm wide tip that was positioned approximately 100 μm away from the cell of interest. The solution was applied for 10 s at a pressure of 4 psi. 10 μM fluorescein was added to the agonist solution to visualize the outflow of the solution from the pencil. NMDAR-mediated responses were isolated by the application of 1 μM tetrodotoxin citrate, 5 μM bicuculline methiodide, 10 μM nifedipine, and 10 μM NBQX (all blockers were from Alomone, except for bicuculline methiodide, which was from Sigma). The responses were significantly diminished by the application of 100 μM D-AP5, an NMDAR blocker (Alomone), and completely abolished by the joint application of 100 μM D-AP5 and 20 mM Mg^2+^. In each slice, responses from only one neuron were recorded. Signals were filtered on-line with a 3 kHz low-pass filter, sampled at 20 kHz, amplified by a Multiclamp 700B amplifier, and acquired with a Digitdata 1550B acquisition card (Molecular Devices). Following off-line filtering at 10 Hz and data reduction by a factor of 100 (performed in Clampfit), the traces were transferred to Microsoft Excel for the calculation of peak amplitude. To express the responses as current density (pA/pF), during each experiment a series of 100-ms-long negative voltage pulses (−25 mV to −5 mV in 5 mV increments) was applied to the voltage clamped cell in the whole-cell mode. Cell capacitance was estimated by two methods: Using a single-exponential fitting of the evoked current relaxation from 80% to 10% of the peak (τ calculation) and by calculating the area under the transient (Q calculation).

### 2.7. Single-Cell [Ca^2+^]_i_ Measurements

The [Ca^2+^]_i_ in cortical neurons was monitored using the ratiometric Ca^2+^ indicator dye Fura-2 acetoxymethyl ester (Fura-2 AM) as previously described [31]. Cells were grown on eight-well chamber slides and loaded with 2 µM Fura-2 AM for 30 min at 37 °C in Hanks Balanced Salt Solution (HBSS) that contained 10 mM HEPES (pH 7.4), 5 mM KCl, 145 mM NaCl, 0.75 mM Na_2_HPO_4_, 10 mM glucose, and 1 mM MgCl_2_ supplemented with 2 mM CaCl_2_ at 37 °C (high Ca^2+^ medium) and then rinsed and left undisturbed for 30 min at 37 °C to allow for de-esterification. Measurements of intracellular Ca^2+^ levels were performed every 1 s at 37 °C using an Olympus Scan^R & Cell^R imaging system that consisted of an IX81 microscope (Olympus, Tokyo, Japan), 10 × 0.40 NA UPlanS Apo objective (Olympus, Tokyo, Japan), and Hamamatsu EM-CCD C9100-02 camera (Hamamatsu Photonics K.K., Hamamatsu City, Japan). Changes in intracellular Ca^2+^ concentration ([Ca^2+^]_i_) in individual neuronal cell bodies are expressed as the F_340_/F_380_ ratio after subtracting background fluorescence and as an area under the curve (AUC). This ratio represents the emission intensities at 510 nm obtained after excitation at 340 and 380 nm. To measure Ca^2+^ response via NMDAR, neurons were stimulated with 100 µM NMDA + 10 µM glycine in the presence of 2 mM CaCl_2_, 5 μM NM, and 30 μM CNQX and in the absence of Mg^2+^. The low Ca^2+^ medium (Ca^2+^-free solution) contained 0.5 mM EGTA in the standard buffer. At the end of all experiments, 50 mM KCl in the presence of 2 mM CaCl_2_ was added to assess which cells were neurons. Cells that responded with rapid, high [Ca^2+^]_i_ rise were identified as neurons and only these cells were analyzed in the experiments. Approximately 60% of cells responded to KCl. Cells that responded with a delay or did not respond at all were assumed to be non-neuronal glial cells, most likely astrocytes. Data processing was performed using Olympus Cell^R software.

### 2.8. Co-Immunoprecipitation and Western Blot

Immunoblotting and co-immunoprecipitation (Co-IP) was performed as previously described [31]. For examination of endogenous STIM-NMDAR interaction, 15-day-old primary cortical neurons grown on Petri dishes were treated for 10 min with 2 mM CaCl_2_ or 0.5 mM EGTA + 2 µM TG in HEPES Buffered Salt Solution (HBSS) and then lysed and homogenized in 1 mL lysate buffer, pH 7.5, that contained 50 mM Tris-HCl, 150 mM NaCl, 0.1% sodium dodecyl sulfate (SDS), 0.5% sodium deoxycholate, 1% NP-40, and 1 mM phenylmethylsulfonyl fluoride supplemented with complete ethylenediaminetetraacetic acid-free protease inhibitor cocktail (Roche). Precleared lysates were incubated overnight at 4 °C on a rocking platform with 30 µL of G-Agarose (Roche) that was pre-incubated earlier for 3 h with 3 µg of antibody (anti-STIM1, ProteinTech Group, Manchester, UK; rabbit STIM2, Alomone Labs, Jerusalem, Israel; rabbit NR2A, Merck Millipore, Darmstadt, Germany; mouse NR2B, Merck Millipore). As a negative control when indicated, lysates were incubated with anti-immunoglobulin G (IgG) antibody (Sigma). The precipitated proteins were then washed three times with repeated centrifugation, eluted in 50 µL of 2× Laemmli Buffer, and subjected to 10% SDS-polyacrylamide gel electrophoresis (PAGE) and western blot analysis with the indicated primary antibodies rabbit STIM1 (1:200, ProteinTech Group), rabbit STIM2 (1:100, Alomone Labs), rabbit NR2A (1:200, Merck Millipore) and rabbit NR2B (1:300, ProteinTech Group) at 4 °C overnight and then with the appropriate horseradish peroxidase-conjugated secondary antibody IgG (1:5000, Sigma) diluted in blocking solution (TBST: 50 mM Tris-HCl [pH 7.5], 150 mM NaCl, and 0.1% Tween 20 plus 5% dry non-fat milk). The immunoreactive bands were developed using a chemiluminescence detection kit (ECL, Promega, Madison, WI, USA). GAPDH was run to normalize the protein loading. The optical density of the bands was estimated using a GS-800 Calibrated Densitometer and Quantity One software (Bio-Rad, Hercules, CA, USA).

### 2.9. Immunocytochemistry

For the immunocytochemical experiments (IF), neurons cultured on coverslips and stimulated for 10 min with 2 mM CaCl_2_ or 2 µM TG in 0.5 mM EGTA were fixed in ice-cold 4% paraformaldehyde and 4% sucrose in phosphate-buffered saline (PBS) for 10 min at room temperature. After permeabilization in 0.1% Triton X-100 and blockade with 2% normal donkey serum (NDS) in PBS for 30 min, an antibodies against rabbit STIM1 (1:50, ProteinTech Group) or mouse STIM1 (1:25, clone CDN3H4, Abnova), rabbit STIM2 (1:50, Alomone Labs), mouse NR2A (1:25, clone E-4, Santa Cruz Biotechnology), mouse NR2B (1:50, clone N59/36, Abcam, Cambridge, UK) and chicken MAP2 (1:500, Invitrogen) diluted in 2% NDS were applied for 2 h at room temperature. The staining was detected using anti-mouse Alexa Fluor 488-, anti-chicken Alexa Fluor 568- and anti-rabbit Alexa Fluor 647-conjugated secondary antibody (Invitrogen) in blocking solution for 45 min at room temperature. To visualize the nuclei of cells, we included the Hoechst 33342 dye (Invitrogen) in the wash. Coverslips were mounted on slides with ProLong Gold Antifade Mountant (Invitrogen). 

### 2.10. Image Processing and Analysis

Images of IF were acquired using a Zeiss LSM 800 confocal microscope with ZEN software. Image processing and analysis was performed using NIH ImageJ software. For each experiment, neurons were always stained in parallel and imaged using identical exposure times and post-acquisition image processing (threshold: 90, ratio: 50%). The processed images were thresholded, and then the Colocalization function was used to obtain merged images of green (NR2A or NR2B), red (STIM1 or STIM2) and blue (nuclei) channels and images of Colocalized points 8-bit to show the co-localization of NR subunits with STIM proteins. Finally, the Manders overlap coefficient was determined to estimate the value of protein co-localization.

### 2.11. Proximity Ligation Assay in Neurons

Neurons, grown on 16-well chamber slides for 17 days, after stimulation with 2 mM CaCl_2_ or 2 µM TG (Sigma) in 0.5 mM EGTA in HBSS for 10 min were immediately fixed in 4% paraformaldehyde in PBS for 10 min at room temperature, and the chambers were removed from the slides. Thereafter cells were subjected to PLA using the Duolink in situ kit (Sigma) according to the manufacturer’s instructions and as we previously described [47]. Briefly, the cells were blocked for 1 h with one drop of Duolink Blocking solution in a humidified chamber at 37 °C and incubated overnight at 4 °C with appropriate combinations of antibodies in Duolink Antibody Diluent solution (40 µL). The antibodies used for the PLA were rabbit anti-STIM1 (1:400, ProteinTech Group) combined with mouse anti-NR2A (1:500, Santa Cruz Biotechnology) or mouse anti-NR2B (1:2000, Abcam) and rabbit anti-STIM2 (1:400, Alomone Labs) combined with mouse anti-NR2A (1:500, Santa Cruz Biotechnology) or mouse anti-NR2B (1:2000, Abcam). After washing with Wash Buffer A, the cells were incubated for 1 h at 37 °C with PLA probes, which are secondary antibodies (Duolink In Situ PLA Probe anti-Mouse MINUS and anti-Rabbit PLUS) conjugated to unique oligonucleotides. Afterward, the samples were incubated with Duolink Ligation-Ligase solution for 30 min at 37 °C, followed by washing in Wash Buffer A and incubation with Duolink Amplification-Polymerase solution for 100 min at 37 °C. Finally, the slides were washed in Wash Buffer B followed by fixation in Duolink Mounting Medium with Dapi and evaluated using an Eclipse 80i fluorescent microscope with a 100× objective (Nikon) and NIH ImageJ software. Representative results are shown from experiments repeated three times. The processed images were thresholded, and the number of in situ PLA signals per cell that corresponded to integrated STIM and NR puncta was quantified using the Particle Analysis function. The settings were kept constant for all of the images throughout the experiments. Quantifications were performed from 15–30 images (*n*) from a minimum of two slides for each culture preparation for every condition (2 mM CaCl_2_ or 2 µM TG with 0.5 mM EGTA), corresponding to 42–64 cells. As a negative technical control, the primary antibodies anti-STIM1, STIM2, NR2A and anti-NR2B were used alone, or both primary antibodies were omitted. These negative controls did not yield any significant PLA signals in either treatment condition.

### 2.12. Statistical Analysis

The statistical analysis was performed using Prism 5.02 software (GraphPad, San Diego, CA, USA). All of the data are expressed as mean ± standard error of the mean (SEM), and differences were considered significant at *p* < 0.05. Statistical significance was assessed using the nonparametric Mann-Whitney U test or one-way analysis of variance (ANOVA) with Tukey’s Multiple Comparison Test as indicated in the legends to the Figures. All of the experiments were performed at least in triplicate.

## 3. Results

### 3.1. NMDA Receptor Antagonists Attenuate TG-Induced SOCE in Neurons

**Figure 1:**
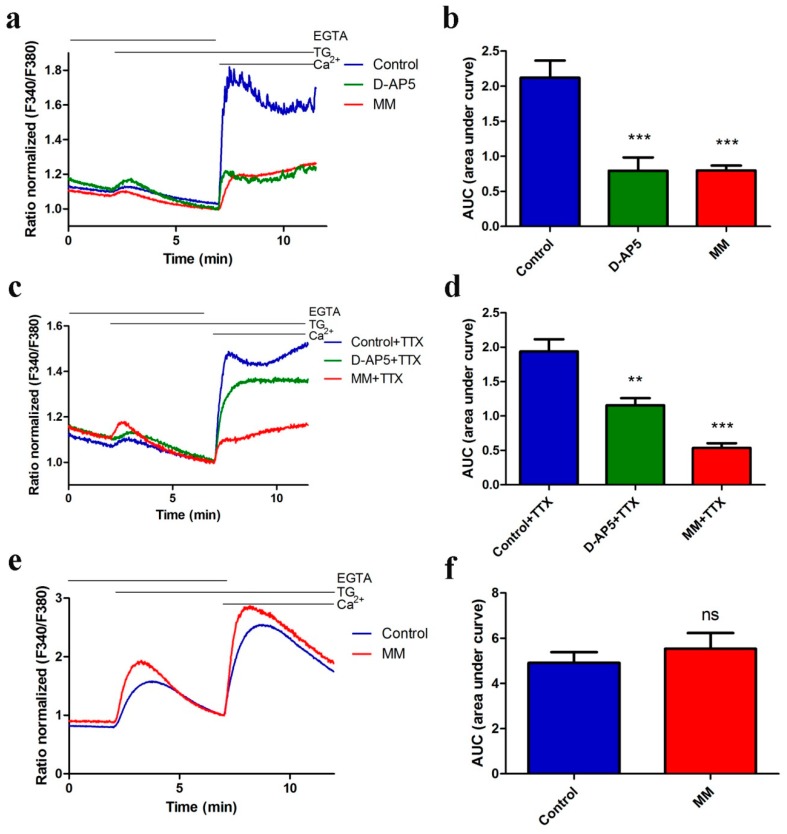
NMDAR antagonists block TG-induced SOCE in rat cortical neurons but not HeLa cells. Average traces of intracellular Ca^2+^ (F340/F380) levels obtained by ratiometric Fura-2AM analysis of neurons in the absence (**a**) or presence of 1 µM TTX (**c**), or in HeLa cells (**e**) treated with 50 µM D-AP5 (green line) or 50 µM MM (red line) and untreated cells (blue line). Measurements were started in a medium with 0.5 mM EGTA, which was then replaced by a medium with 0.5 mM EGTA and either 2 µM TG + 50 µM D-AP5 or 2 µM TG + 50 µM MM. Finally, 2 mM CaCl_2_ was added to the medium to trigger nSOCE with either 50 µM D-AP5 or 50 µM MM. F340/F380 values just before the addition of Ca^2+^ were normalized to the same values (1). (**a**–**d**) The data represent *n* = 28 (Control), *n* = 12 (D-AP5), *n* = 20 (MM), *n* = 15 (Control + TTX), *n* = 19 (D-AP5 + TTX) and *n* = 18 (MM + TTX) independent experiments that were conducted on five different primary cultures, corresponding to 1160, 513, 780, 336, 390, and 710 analyzed cells that responded to KCl-induced membrane depolarization, respectively. (**e**–**f**) The data represents 17 independent measurements conducted in four different experiments corresponding to 1333 for control and 1315 for MM treated cells, respectively. (**b**,**d**,**f**) Summary data of panels (**a**,**c**,**e**) presented as the area under the curve (AUC) showing Ca^2+^ influx, which was calculated from the moment immediately before adding Ca^2+^ from minutes 7 to 11; ns (not significant), ** *p* < 0.01, *** *p* < 0.001 significantly different compared with the control (Mann-Whitney U test). Data are expressed as the Delta Ratio (±SEM).

We explored if NMDARs participate in the mechanisms underlying TG-induced nSOCE using the Ca^2+^ addback assay. Primary cultures of cortical neurons were first treated with the SERCA pump inhibitor thapsigargin (TG) in the presence of a Ca^2+^ chelator (ethylene glycol tetraacetic acid; EGTA) to deplete Ca^2+^ in the ER. We then added Ca^2+^ back to measure Ca^2+^ influx from the extracellular medium using a Ca^2+^ Fura-2AM fluorescence probe in the absence or presence of specific NMDAR antagonists: either D-AP5 (selective competitive NMDAR antagonist) or memantine (open channel NMDAR blocker, MM) added at the beginning of the experiments. Figure 1a shows both antagonists inhibited nSOCE. Blocking NMDAR by 50 μM D-AP5 or MM reduced SOCE approximately by 63% compared to the Ca^2+^ response observed in the absence of these drugs. This result is reflected by a statistically significant decrease of area under the curve (AUC) values from 2.12 to 0.795 for D-AP5 (green bar) and 0.799 for MM treated cells (red bar) (Figure 1b). The AUC values were calculated from the moment immediately before the addition of extracellular Ca^2+^ for 4 min (time period of 7–11 min).

We cannot exclude that the addition of 2 mM Ca^2+^ induces synaptic activity, causing Ca^2+^ influx also via NMDA and AMPA receptors. To eliminate the possible effect of synaptic activation on nSOCE, we repeated the above experiments in the presence of 1 μM tetrodotoxin (TTX), which inhibits activity-dependent synaptic transmission in neurons. In the presence of TTX and D-AP5, we observe SOCE inhibition by 40% (Figure 1c,d). It is a 23% smaller inhibitory effect compared with D-AP5 alone but still statistically significant (** *p* < 0.01). In contrast, the presence of TTX and memantine caused even a greater reduction of nSOCE by 72% compared to 63% in the absence of TTX (Figure 1c,d). This indicates that the inhibitory action of NMDAR antagonists on nSOCE is not related to the synaptic activities. 

To eliminate the possibility that inhibitory effect of the NMDAR antagonists on nSOCE occurs through direct inhibition of STIM1, STIM2 or Orai proteins, we examined these responses in HeLa cells, since they do not express endogenous NMDARs [48], in the presence of one NMDAR antagonist. As shown in Figure 1e,f, 50 µM MM did not affect SOCE in HeLa cells. These results suggest that attenuating nSOCE by NMDA antagonists as shown in Figure 1a–d requires the presence of NMDARs. Altogether, we conclude that NMDARs do contribute to the mechanisms of nSOCE in the presence of TG as observed in cortical neurons.

### 3.2. SOCE Inhibitor SKF96365 Enhances NMDA-Stimulated [Ca^2+^]_i_

**Figure 2:**
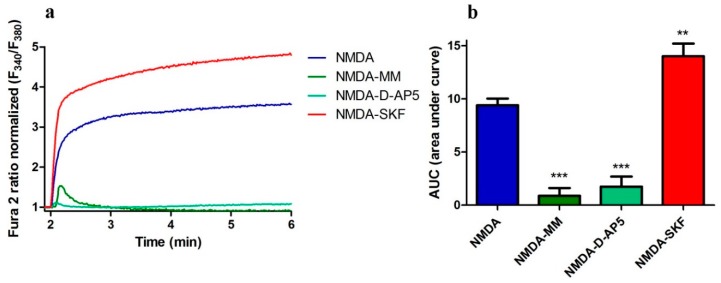
SKF96365 increases NMDA-induced Ca^2+^ levels. (**a**) Analysis of the [Ca^2+^]_i_ induced by NMDA (100 μM) and glycine (10 μM) in the presence of 5 μM nimodipine, 30 μM CNQX, and 50 μM MM; 50 μM D-AP5 or 30 μM SKF96365 (SKF) based on ratiometric measurements with Fura2-AM. F_340_/F_380_ values just before adding the NMDAR agonist were normalized to the same values (1). The data represent *n* independent experiments that were conducted on four different primary cultures corresponding to 516 (NMDA, *n* = 13), 305 (NMDA-MM, *n* = 10), 245 (NMDA-DAP, *n* = 10) and 624 (NMDA-SKF, *n* = 19) analyzed cells. (**b**) Summary of data from (**a**) shown as area under the curve (AUC), which was calculated from the moment immediately before the addition of NMDA. ** *p* < 0.01; *** *p* < 0.001 significantly different compared with NMDA (ANOVA followed by Tukey’s Multiple Comparison Test).

Since NMDAR antagonists decreased nSOCE, we next examined whether a SOCE inhibitor affects [Ca^2+^]_i_ induced by NMDA receptor stimulation using the selective agonists, glycine, and NMDA. Cells were incubated in HBSS that contained 2 mM Ca^2+^ and no Mg^2+^. To eliminate the confounding effects of L-type VGCCs and AMPARs, we added their antagonists, nimodipine and CNQX, to all respective experiments. First, we determined if Ca^2+^ level elevations induced by NMDA in rat cortical neurons includes Ca^2+^ entry via NMDAR in the presence of its inhibitors (D-AP5 or MM). Next, cell stimulation with 100 µM NMDA in the presence of glycine elicited a robust elevation in cytosolic Ca^2+^ signals (blue line), which was suppressed by both NMDAR antagonists (81% by D-AP5 and 90% by MM) as shown in Figure 2. As expected, this result shows that NMDARs under our conditions in cortical neuronal cultures are expressed and mediate the major Ca^2+^ influx induced by NMDA stimulation. To investigate the contribution of SOCE proteins to these NMDA-induced Ca^2+^ responses, we applied the SOCE inhibitor SKF96365 together with the NMDAR agonists. SKF96365 can inhibit STIM1-mediated SOCE [4,49]. Adding SKF96365 to this treatment enhanced Ca^2+^ levels evoked by applying NMDA by 49% (Figure 2). The effect of SOCE inhibitor on NMDA-induced Ca^2+^ level suggests the involvement of STIM proteins.

### 3.3. Downregulation of STIM1 or STIM2 Enhance NMDA-Induced Ca^2+^ Signals

**Figure 3:**
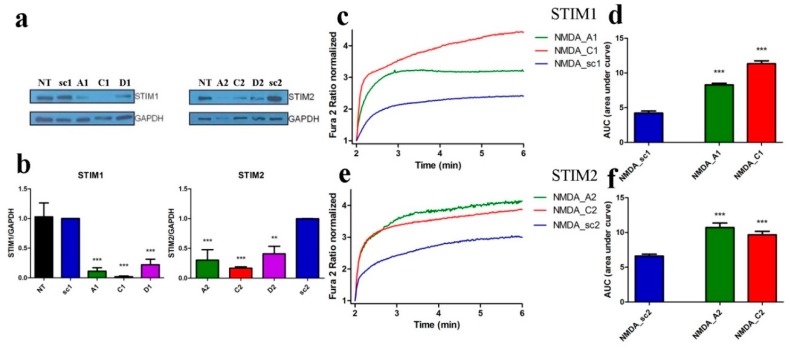
shSTIM1 and shSTIM2 increase NMDA-induced Ca^2+^ responses. (**a**) Western blot analysis of STIM1 and STIM2 protein levels using anti-STIM1 and anti-STIM2 antibodies in cortical neurons transduced with lentiviruses expressing different shRNA sequences directed against RNA for STIM1 (A1, C1, or D1), for STIM2 (A2, C2, or D2) or the control sequence shRNA (sc1, sc2). GAPDH served as reference. (**b**) Results of quantitative WB analysis of cell lysates obtained from neurons transduced as in (**a**). Each column shows the mean ± SEM of three independent transductions. Statistical analysis performed by ANOVA, followed by Tukey’s Multiple Comparison Test. NT, non-transduced control, ** *p* < 0.01; *** *p* < 0.001. (**c**–**f**) NMDAR agonists-induced [Ca^2+^]_i_ responses increased when expression of STIM1 (**c**,**d**) and STIM2 (**e**,**f**) is silenced by shA and shC compared to neurons transduced with shsc control plasmid. F_340_/F_380_ values just before adding NMDAR agonists normalized to the same values (1). Data represent *m* number of analyzed cells in *n* independent experiments that were conducted on three different primary cultures (NMDA_sc1, *m* = 89, *n* = 5), (NMDA_A1, *m* = 98, *n* = 5), (NMDA_C1, *m* = 96, *n* = 7), (NMDA_sc2, *m* = 104, *n* = 6), (NMDA_A2, *m* = 60, *n* = 7), and (NMDA_C2, *m* = 92, *n* = 9). (**d**,**f**) Summary of graphs (**c**,**e**) shown as an area under the curve (AUC). *** *p* < 0.001 significantly different compared with NMDA_sc (ANOVA followed by Tukey’s Multiple Comparison Test).

Next, we investigated if STIM proteins contribute to [Ca^2+^]_i_ in neurons after NMDAR activation. We silenced the expression of Stim1 or Stim2 genes in neurons by transducing cultured neurons with lentiviruses carrying a GFP tag and three shRNA sequences against each Stim gene. The efficiency of gene knockdown varied among different shRNAs based on STIM1/STIM2 protein levels on Western blots (Figure 3a).

When compared with neurons transduced with control shRNA sequences (scramble; sc1, sc2), all constructs efficiently decreased STIM1 protein level by 89%, 98%, and 78% for plasmids A1, C1 and D1, respectively and STIM2 protein by 70%, 83%, and 59% for plasmids A2, C2, and D2, respectively (Figure 3a,b). Two constructs that best silenced STIM1 and STIM2 proteins (A and C) were used for single-cell [Ca^2+^]_i_ measurements. In all cases, downregulating STIM1 (A1, C1) and STIM2 (A2, C2) increased the AUC in the cytoplasm mediated by NMDA by 96%, 169% (Figure 3c,d) or 63%, 47% (Figure 3e,f) respectively, compared to the line transduced with control plasmids (sc1, sc2). Thus, reduction of STIM protein levels increased the NMDA-induced [Ca^2+^]_i_ response. 

### 3.4. Overexpressing STIM1/2 Suppresses NMDA-Induced [Ca^2+^]_i_ Elevations 

**Figure 4:**
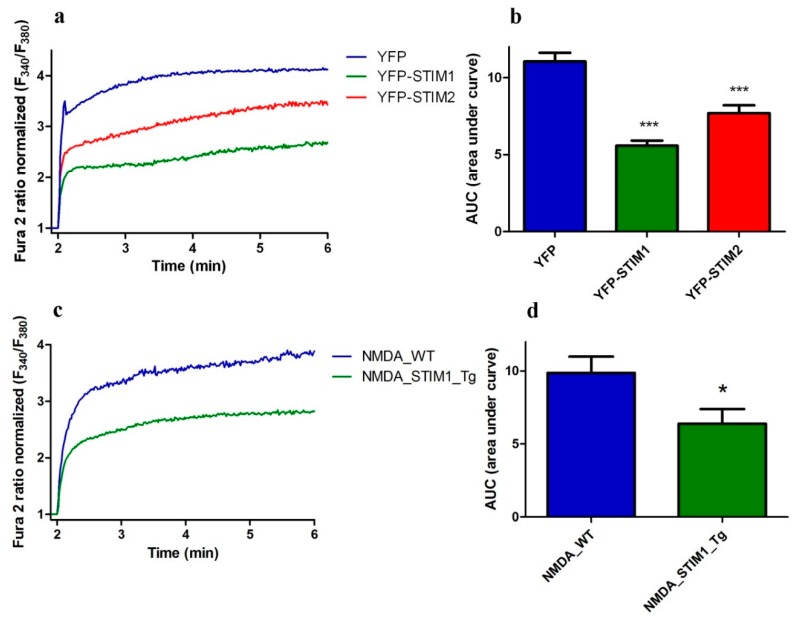
Neurons with overexpressed STIM1 and STIM2 exhibit lower NMDA-induced [Ca^2+^]_i_. (**a**) Analysis of NMDA and glycine-induced [Ca^2+^]_i_ in the presence of nimodipine and CNQX based on ratiometric measurements with Fura2-AM in neurons overexpressing YFP-STIM1, YFP-STIM2, or YFP. F_340_/F_380_ values immediately prior to adding the NMDAR agonist were normalized to the same values (1). Data represent *n* independent experiments that were conducted on *h* different primary cultures corresponding to 71 (YFP, *n* = 11, *h* = 4), 104 (YFP-STIM1, *n* = 16, *h* = 6) and 81 (YFP-STIM2, *n* = 16, *h* = 6) analyzed cells. (**b**) Summary of data from (**a**) shown as an AUC. *** *p* < 0.001 significantly different compared with YFP (ANOVA followed by Tukey’s Multiple Comparison Test). (**c**) Analysis of [Ca^2+^]_i_ induced by NMDA and glycine in the presence of nimodipine and CNQX based on ratiometric measurements with Fura2-AM in neurons from Tg(STIM1)Ibd (Tg) or control (wild type, WT) mice. F_340_/F_380_ values immediately prior to adding the NMDAR agonist were normalized to the same values (1). The data represent *n* independent experiments that were conducted on four different primary cultures, corresponding to 196 (NMDA_WT, *n* = 20) and 183 (NMDA_STIM1_Tg, *n* = 17) analyzed cells. (**d**) Summary of data from (**c**) shown as AUC. * *p* < 0.05 significantly different compared with NMDA_WT (Mann–Whitney U test).

To further investigate whether the NMDA-stimulated [Ca^2+^]_i_ response is sensitive to STIM proteins, we monitored [Ca^2+^]_i_ in neurons with increased levels of STIM1 and STIM2 protein expression using transfection of cortical neurons with plasmids encoding YFP-tagged STIM1 or YFP-tagged STIM2. As shown in Figure 2a and Figure 4a, 100 µM NMDA + glycine induced a prolonged Ca^2+^ increase characterized by a sharp increase in signal and a sustained plateau (blue line). Overexpression of either STIM1 or STIM2 reduced cytosolic [Ca^2+^] after NMDA stimulation by 50% and 30%, respectively (Figure 4a,b).

To further confirm the role of STIM proteins in NMDAR activity, we compared the response to NMDA stimulation in neurons from wild-type mouse with that from our newly generated STIM1-overexpressing Tg(STIM1)Ibd mice [23]. After NMDA activation, primary cortical cultures from Tg(STIM1)Ibd mice showed 35% decrease in Ca^2+^ responding compared to cultures of wild type neurons (Figure 4c,d), which is consistent with our results obtained with transient overexpression of YFP-STIM1 (Figure 4a,b). These findings are in agreement with the data shown in Figure 3. Taken together, our results suggest that STIM1 and STIM2 negatively control the NMDA-evoked Ca^2+^ elevations in rat cortical neurons.

Using acute brain slices prepared from STIM1-overexpressing transgenic mice, we performed electrophysiological experiments to assess whether STIM1 contributes to NMDAR function. NMDAR-dependent currents were recorded from visually identified layer V pyramidal neurons of the cortex. Standard ACSF without Mg^2+^ ions and supplemented with 10 µM glycine was used. After obtaining stable whole-cell configuration, the agonist solution (100 µM Na-glutamate in Mg^2+^-free ACSF) was applied locally above the patched neuron with the use of an automated perfusion system (ValveLink 8.2, AutoMate Scientific, Berkeley, CA, USA) for 10 s. To isolate NMDAR-mediated currents, action-potential dependent activity was blocked by the application of 1 µM tetrodotoxin, and AMPARs and VGCCs were blocked by 10 µM NBQX and 10 µM nifedipine, respectively. Application of NMDA evoked robust inward currents that were significantly diminished by an NMDAR blocker, D-AP5 (100 µM), and almost entirely blocked by joint application of 100 µM D-AP5 and 20 mM Mg^2+^ (Figure S1a). The responses were quantified both as peak current amplitude (pA) and as current density (pA/pF; to normalize for differences in cell size). No significant changes in either of the two parameters were detected between neurons from wild-type and *Tg*(Stim1)Ibd transgenic mice (Figure S1a–c). Therefore, we concluded that overexpression of STIM1 in layer V pyramidal neurons of the cortex had no detectable impact on the function of NMDA receptors that was measured with the patch-clamp technique, which is in contrast with Fura-2 imaging data. This discrepancy might stem from the fact that in the latter approach, fluxes of the Ca^2+^ ions alone were measured, while in patch-clamp experiments, mostly Na^+^ contributed to the measured currents. 

### 3.5. STIMs Directly Interact with the NR2 Subunit Using PLA 

**Figure 5:**
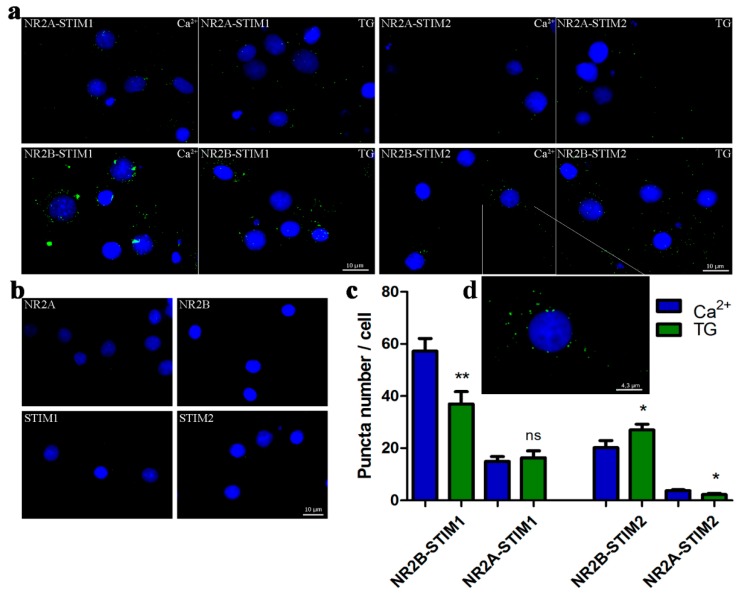
The interaction between endogenous STIM1/STIM2 and NR2A/NR2B occurs in situ. (**a**) Proximity ligation assay between STIM1 and NR2B, STIM1 and NR2A, STIM2 and NR2B, and STIM2 and NR2A before and after store depletion by TG/EGTA observed by fluorescent microscopy. Neurons were also counter-stained with the nuclear marker Hoechst dye (blue). The PLA signal, recognized as a fluorescent green dot, shows the close proximity of STIM and NR2 antigens. (**b**) No signals were observed when one of the primary antibodies was omitted (either anti-NR2A or NR2B or STIM1 or STIM2) that demonstrates the specificity of the detection assay and used antibodies. Scale bar, 10 µm for each panel. (**c**) Quantification of the complexes detected by PLA. Bars represent averages from 15–30 (*n*) images taken in three independent experiments, corresponding to 42–64 cells ± SEM. The quantification of PLA signals was performed using ImageJ software to analyse neurons. * *p* < 0.05; ** *p* < 0.01; ns, not significant compared with the control (Mann–Whitney U test). (**d**) The picture shows the higher magnification of one neuron from (**a**) to better visualize the PLA signals and their localization in the cell.

Since our results suggest STIM proteins contribute to NMDA-induced Ca^2+^ levels, we investigated the nature of the interaction between STIM and NMDAR proteins using complementary approaches. To analyze the formation of endogenous STIM-NR2 complexes in situ, we performed the Proximity Ligation Assay (PLA) using established methods [47,50]. In brief, PLA uses primary antibodies against analyzed proteins of interest and secondary antibodies from other species targeting primary antibodies coupled to oligonucleotides. If examined antigens (here STIMs and NR2s) are in close proximity the oligonucleotides can be ligated into a closed circle, which is then amplified and detected as a fluorescent dot. PLA allows not only visualization of protein–protein interaction in situ, but also the semi-quantitative analysis of their interaction [51]. Neurons treated with either 2 mM CaCl_2_ (control) or TG/EGTA were fixed, permeabilized and probed with anti-NR2A or anti-NR2B, and anti-STIM1 or anti-STIM2 primary antibodies. As shown in Figure 5a, we observed dot-like green positive signals in all cells analyzed under control condition (high Ca^2+^ medium). These data indicate a proximity between NR2 and STIMs at a maximum distance of about 40 nm [50]. The approximate average relative number of NR2B-STIM1 complexes per cell was 57.2 ± 4.906 and 3.78 ± 0.346 of NR2A-STIM2 complexes (Figure 5c). The green fluorescent dots were localized mostly in cell bodies (Figure 5a,d). We found no signals when applying only one primary antibody, followed by incubation with the two oligonucleotide-conjugated secondary antibodies, rabbit-PLUS and mouse-MINUS (Figure 5b). After Ca^2+^ store depletion by TG/EGTA, the number of NR2B-STIM2 complexes increased by 33% compared with high Ca^2+^ medium (Figure 5c), while NR2B-STIM1 and NR2A-STIM2 complexes decreased by 35% and 41%, respectively. We did not detect any significant changes in the number of NR2A-STIM1 complexes.

### 3.6. STIM Proteins Co-Localize with NMDAR Subunits Using Immunofluorescence 

**Figure 6:**
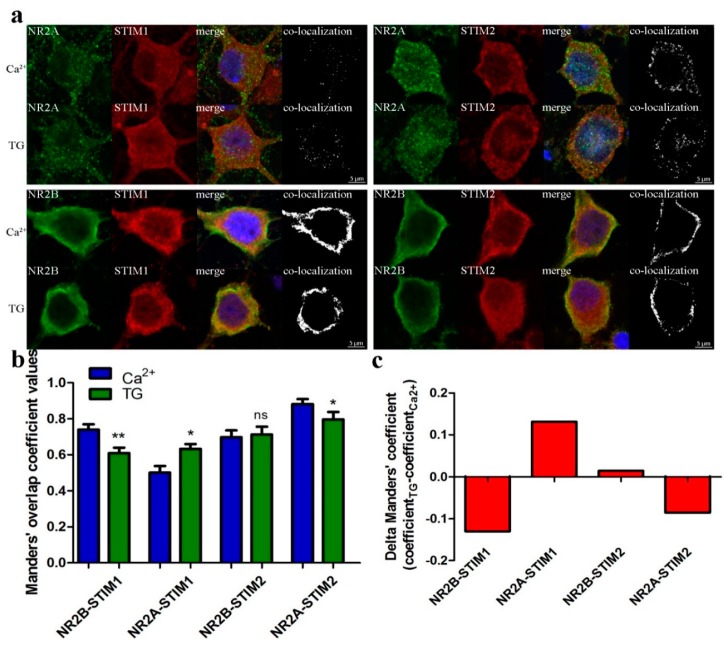
Endogenous STIMs co-localize with NMDAR2. (**a**) Representative confocal images of Ca^2+^ or TG/EGTA-treated neurons fixed and stained with anti-NR2A or NR2B antibody (shown in green, 1st panels), with anti-STIM1 or anti-STIM2 (shown in red, 2nd panels) and with anti-MAP2 to identify neurons for analysis (not shown for clarity). (Blue) Nuclei were stained with Hoechst. The 3rd columns show the merged images of green, red and blue channels. Co-localization of NR subunits with STIM is shown in the 4th columns (white). All images are taken from a single slice from the middle of the cell. Scale bar, 5 µm for each panel. (**b**,**c**) Results of co-localization analysis of NRs with STIMs in soma of neurons treated as in (**a**). Bar graph depicting the quantification of the average value of STIM with NR co-localization coefficient according to Manders (**b**) or the difference between this coefficient value 10 min after TG treatment and before store depletion (in the presence of 2 mM Ca^2+^) (**c**). * *p* < 0.05; ** *p* < 0.01; *ns*, not significant compared with the control (Mann-Whitney U test). Bars represent the average of cultures from three animals; 35–55 cells per each condition (Ca^2+^ or TG/EGTA).

We next examined whether endogenous STIM1 or STIM2 proteins co-localized with NMDAR2 subunits (NR2A and NR2B). Cortical neurons cultured under control conditions (2 mM Ca^2+^) or after Ca^2+^ store depletion by TG/EGTA were fixed, permeabilized and probed with antibodies against STIM proteins and NMDAR subunits. The neuronal marker MAP2 and nuclear Hoechst indicator dye were used to identify the neuronal cells and their nuclei. By analyzing immunostained proteins by confocal microscopy, we found clear co-localization of endogenous STIM1 and STIM2 proteins with both NMDAR subunits. The value of protein co-localization was determined using the Manders overlap coefficient [52]. The highest co-localization occurred between NR2A and STIM2 proteins, while NR2A and STIM1 showed the least co-localization (Figure 6). Treating neurons with TG/EGTA increased co-localization of NR2A and STIM1 by 22.3%, whereas it abolished co-localization of NR2B with STIM1 and NR2A with STIM2 by 17.5% and 9.7%, respectively, as compared to overlap before nSOCE induction (Figure 6b,c). Thus, co-localization of STIM1 and NMDAR2 subunits may require Ca^2+^ store depletion. Since we found no changes in Manders overlap coefficient value for NR2B-STIM2 co-staining, these data indicate that the formation of STIM2 complexes with NR2B subunits occurs independent of the SOCE process and ER Ca^2+^ content. Interestingly, both the location and intensity of co-staining of cortical neurons with antibodies recognizing STIM proteins differed depending on NMDAR subunits. For instance, STIM2 showed strong co-localization with NR2B near the PM and possibly in the ER. Our immunofluorescence data show the same vector of changes in co-localization as our estimated changes using PLA (Figure 5).

### 3.7. STIM-NMDAR Subunits Interact by Co-Immunoprecipitation

**Figure 7:**
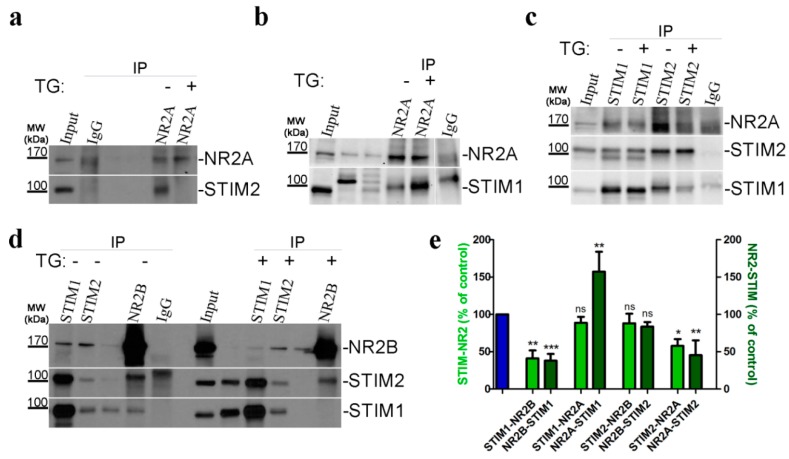
Endogenous STIMs co-immunoprecipitate with NMDAR2. (**a**–**d**) Representative WBs from Co-IP experiments investigating the interaction between endogenous STIM1, STIM2 and NR2A, NR2B subunits, demonstrating a change in the interaction upon SOCE activation by TG (TG; +) compared with control neurons treated with 2 mM Ca^2+^ (TG; −). Neuronal lysates (Input) and eluted fractions (immunoprecipitates; IP) were separated on 10% sodium dodecyl sulfate gels, analyzed by WB and stained with the corresponding antibody anti-STIM1, STIM2, NR2A, and NR2B (as indicated on the right) as described in “Methods and Materials” section. Anti-IgG antibody was used as a negative control. WB analysis of 40 µg of cell lysate inputs is shown. Molecular weights of the markers run on the same gel are indicated on the left (in kDa). (**b**,**d**) Unlabeled bands are irrelevant to this experiment. Unspecific IgG band is visible. (**c**) The middle panel shows the WB stained with STIM2 protein after stripping the membrane blotted with anti-STIM1 antibody, indicated with double bands here. (**e**) Pooled data shows a significant change in interaction between STIM proteins and NMDAR subunits after TG treatment. Histogram represents the quantification of STIM-NR (light-green columns) or NR-STIM (dark-green columns) association in neurons incubated in TG compared to neurons incubated in 2 mM Ca^2+^ (blue column). Bands of co-immunoprecipitates were analyzed densitometrically and normalized to the level of the loading control (i.e., bands obtained after WB with the antibody used for immunoprecipitation). The results are expressed as a percentage of control (i.e., protein association in 2 mM Ca^2+^). Bar graphs are mean ± SEM of at least three independent experiments. * *p* < 0.05; ** *p* < 0.01; *** *p* < 0.001 significantly different compared with control; *ns*, not significant compared with the control (Mann–Whitney U test).

To confirm the physical association between endogenous STIM proteins and endogenous NMDAR subunits, we prepared total homogenates of cultured cortical neurons for co-immunoprecipitation (co-IP) experiments, followed by western blot (WB) analysis. Immunoprecipitates with anti-STIM1 or anti-STIM2 were analyzed by WB with either anti-NR2A (Figure 7c) or anti-NR2B (Figure 7d). The presence of these NMDA subunits was detected and verified by reverse immunoprecipitation with anti-NR2A or anti-N2B antibody. In such immunoprecipitates, we also detected the presence of STIM2 (Figure 7a,d) and STIM1 (Figure 7b,d).

To provide further mechanistic insight, we investigated whether SOCE and STIM activation following Ca^2+^ store depletion modulates the binding between STIM proteins and NMDR subunits. We detected no change in the association between STIM1 and NR2A (Figure 7c) or STIM2 and NR2B (Figure 7d) or after depleting intracellular Ca^2+^ stores by TG (Figure 7e, light-green bars). We also found greater binding between NR2A and STIM1 using co-immunoprecipitation (Figure 7b,e, dark-green bars). We also demonstrated a decrease in binding between NR2B and STIM1 (Figure 7d) and NR2A to STIM2 (Figure 7c) after TG administration (Figure 7e, light-green bars). Figure 7d also shows the reverse co-immunoprecipitation of NR2B with STIM1 and STIM2. We observed that neurons after TG treatment have a partial disruption of the NR2B-STIM1 interaction, but not NR2B-STIM2 (Figure 7e, dark-green bars). TG treatment significantly attenuated the formation of NR2A-STIM2 complexes (Figure 7a) as well the STIM2-NR2A association (Figure 7c), demonstrating the interaction (Figure 7e, dark-green vs. light-green column). In all co-IP experiments, we observed either a very weak or no interaction between the control IgG and any protein of interest.

The co-IP data support our PLA and immunofluorescence co-localization results. Taken together, our results demonstrate the existence of protein complexes formed by STIM1-NR2A, STIM1-NR2B, STIM2-NR2A and STIM2-NR2B. Some of these complexes are sensitive ER Ca^2+^ levels. Depleting Ca^2+^ stores by TG/EGTA dispersed the complexes formed between STIM1-NR2B and between STIM2-NR2A (Table S1). In Table S1, we summarize the findings presented in Figure 5, Figure 6 and Figure 7, which identifies these NMDAR subunits as novel STIM1 and STIM2 interacting proteins.

## 4. Discussion

Our study reveals a previously unidentified, direct link between NMDARs and STIM proteins. To uncover first the mechanisms underlying NMDA-dependent effects on SOCE, we characterized Ca^2+^ signaling induced by depleting Ca^2+^ stores in the presence of NMDAR inhibitors in cultured cortical neurons. We found that two NMDAR antagonists, D-AP5 and memantine, significantly reduced Ca^2+^ influx into rat cortical neurons from the extracellular space during our nSOCE protocol. We concluded that NMDARs participate in Ca^2+^ influx during ER refilling. However, MK-801, another NMDAR antagonist, did not have a significant impact on nSOCE in mouse cortical neurons [53]. We suspect this prior result arose through different NMDAR antagonists and SOCE induction protocols. Gonzalez-Sanchez et al. measured SOCE and performed Ca^2+^ store depletion in a medium containing physiological Ca^2+^ concentrations [53], while we used the more common Ca^2+^ re-addition protocol [45,54]. Yet, MK801 did inhibit SOCE in human T-lymphocytes under the conditions of Ca^2+^ re-addition protocol [55]. Our results show that the increase in Ca^2+^ after its addition can be mainly attributed to SOCE, but an additional small influx of Ca^2+^ is also possible due to the activation of synapses. However, after blocking action-potential driven neuronal activity by TTX, we still observe the inhibitory effect of D-AP5 on SOCE, although not such a large one (about 20% smaller). On the other hand, TTX seems to have no effect on the inhibitory activity of memantine. The differences between the blocking effects of both inhibitors may be due to different sites of their binding to the NMDAR—D-AP5 binds to the agonistic site of NR2 subunit (such as glutamate) and memantine acts as a non-competitive antagonist whose binding site is within the ion channel pore region. The above results support our hypothesis that NMDAR is involved in SOCE.

Ca^2+^ influx into neuronal cells depends on NMDARs, AMPARs, VGCCs, as well as SOCE that trigger Orai/TRPs channels and STIM proteins [20,21,34,35,36]. The role of NMDARs in nSOCE remains unclear. Activating NMDARs induces Ca^2+^ release from the ER in presynaptic hippocampal neurons [32,56,57,58], causing the ER to refill with Ca^2+^ from external sources [58,59]. Synaptic NMDAR stimulation can activate nSOCE to contribute to synaptic plasticity, such as long term potentiation (LTP), but this process is insensitive to the L-type Ca^2+^ channels inhibitors, verapamil and nicardipine [32]. We showed that AMPAR antagonists inhibit nSOCE in cortical neurons [31]. We speculated that nSOCE entails a more complex process then SOCE in non-excitable cells, where it constitutes the main Ca^2+^ entry into the cell. 

Assuming that NMDARs contribute to calcium influx in nSOCE, we examined the effect of a STIM-mediated SOCE inhibitor on NMDA-induced Ca^2+^ influx. SKF96365 (30 µM) increased the amplitude of [Ca^2+^]_i_ induced by NMDA. Using different SOCE inhibitors, such as 30 µM 2-APB, 3 µM SKF96365 and 100 µM La^3+^, Baba et al. found altered slow, but not fast, exponential [Ca^2+^]_i_ decay coefficient of NMDA responses [32]. We suspect different protocols underlie this discrepancy. In their studies, inhibitors were applied a few minutes prior to NMDA exposure, while we added our inhibitor together with NMDA. We also posit that these differences may also occur based on the cell type used. For instance, SOCE inhibitors showed inhibitory effect on Ca^2+^ responses in hippocampal neurons but not in granular neurons [32]. 

We note that commonly used SOCE inhibitors are not highly specific [60] with only a few that target STIMs, as ML-9 and SKF96365 [4,11,49]. Unfortunately, ML-9 also modulates NMDAR function by lowering NMDAR-mediated and miniature excitatory post-synaptic NMDA currents in neurons [61]. Since SKF96365 affects NMDA-induced intracellular Ca^2+^ levels (Figure 2), we speculated that STIMs could also later NMDAR activity. To test our hypothesis and characterize the role of STIM1 and STIM2 in NMDA-induced [Ca^2+^]_i_, we manipulated *STIMs* gene expression in cultured cortical neurons. Downregulating either *STIM* gene expression by shRNAs elevated NMDAR-mediated [Ca^2+^]_i_. In turn, the transient overexpression of YFP-STIM1 or YFP-STIM2 significantly attenuated NMDA-induced [Ca^2+^]_i_ elevation compared to that by YFP expression. Overexpressing STIM1 in mouse cortical neurons from Tg(STIM1)Ibd mice [23] suppressed intracellular Ca^2+^ levels after NMDA treatment (Figure 4). Taken together, these results suggest that STIM proteins help regulate NMDAR function. 

The strongest support for this conclusion came from the proximity ligation assay, co-immunolocalization and co-immunoprecipitation experiments performed either in situ or in vitro. These experiments uncovered the physical interaction between endogenous STIM1 or STIM2 with the NMDAR2 subunits, NR2A and NR2B. Some interactions appeared sensitive to the Ca^2+^ level in ER. We hypothesize that emptying Ca^2+^ from ER stores not only facilitates STIM-Orai1 interaction but also diminishes the formation of hetero-complexes, mainly composed of STIM1-NR2B and STIM2-NR2A. We suspect that STIM2 remains in a complex with NMDAR. After store depletion STIM1 translocates to plasma membrane increasing interaction with NR2A, which displaces STIM2 from complexes with NR2A. Our immunofluorescence data indicates the presence of NR2B mainly in the ER, so store depletion produces the exit of STIM1 from the ER, as indicated by a decreased interaction between STIM1 and NR2B. Previous reports demonstrated that in neurons other channel proteins, such as AMPAR [30,31] or TRPs [25,62] couple or interact with STIMs to modulate SOCE signaling in neurons. Inhibitory regulation and physical coupling can occur between STIM1 and Ca_v_1.2 [27,28,63]. In hippocampal neurons, depolarization by glutamate activates Ca^2+^ influx through NMDARs and L-type VGCCs, to trigger the release of Ca^2+^ from the ER and activate STIM1 [28]. Changes in ER calcium and growth in dendritic spine ER contents due to inhibiting VGCCs by STIM contribute to structural plasticity of dendritic spines [64]. These findings indicate that NMDAR activation activates STIM1 to directly control the structural plasticity of L-type VGCC-dependent dendritic spines [28]. We postulate that STIMs can also modulate NMDAR-mediated Ca^2+^ signaling by directly interacting with NMDARs. Nevertheless, we cannot exclude the possibility that STIM does not directly inhibit NMDARs, but indirectly inhibits it by inhibiting VGCC. However, this seems less likely in our case, because in the experiments the VGCCs were blocked by an inhibitor.

Whole-cell NMDAR-dependent current measurements by electrophysiology revealed no effect of STIM1 overexpression in neurons from transgenic mice (Figure S1). This difference likely arises from the different type of ions measured. Fura-2 AM based imaging allowed to measure only Ca^2+^ fluxes. In contrast, patch-clamp experiments also rely on Na^+^, in addition to Ca^2+^, as primary carrier of the current. The signal source also drives our observed differences between the imaging and electrophysiological data. Even though the electrophysiological signal comes from both cell bodies and neuronal processes, we only quantified the Ca^2+^ imaging signal from cell bodies. Although SOCE inhibitors can inhibit NMDA-induced [Ca^2+^]_i_, the same drugs do not inhibit NMDAR currents using electrophysiology [32]. Further studies will resolve whether the STIM1-NMDAR interaction depends on their neuronal cell localization. 

**Figure 8:**
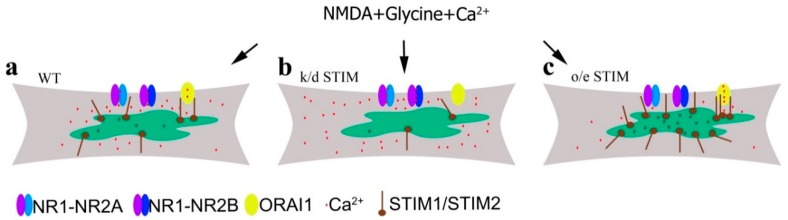
Proposed mechanism of changes in intracellular Ca^2+^ level induced by NMDA in the presence of glycine (without Mg^2+^) under the influence of different STIM protein expression. (**a**) In wild-type neurons with normal STIM protein expression, Ca^2+^ influx occurs via NMDAR and Orai channels. (**b**) After STIM knockdown (k/d), Ca^2+^ influx through the Orai channel is blocked and [Ca^2+^]_i_ is increased due to Ca^2+^ influx via NMDAR and Ca^2+^ retention in the cytosol and its failure to enter the ER. (**c**) Overexpressing STIM (o/e) inhibits NMDA-induced Ca^2+^ influx into the cytosol and increases Ca^2+^ storage in the ER.

STIM and NMDAR abnormalities have been implicated in the pathogenesis of neurodegenerative diseases [20,21]. However, the links between these proteins under normal and disease conditions remain unclear. Overstimulating NMDARs by glutamate or NMDA leads to a prolonged increase in intracellular Ca^2+^ concentration and Ca^2+^ overload. This mechanism is proposed to be the main cause of neuronal death in neurodegenerative diseases associated with excitotoxicity, e.g., HD and AD [41,42,43,65]. To limit neuronal cell death in neuropathological conditions, we must employ strategies to attenuate intracellular Ca^2+^ overload. We propose to target the mechanism that links NMDAR and STIM proteins in cortical neurons (Figure 8), as stimulating NMDARs induces Ca^2+^ influx from the extracellular space and efflux from the ER [28,56,57,58]. Low ER Ca^2+^ content activates STIMs and promotes SOCE [58,59] (Figure 8a). We posit that downregulating STIMs will inhibit SOCE and either disturb or halt Ca^2+^ influx to the ER (Figure 8b) as in Purkinje neurons [25]. This process should retain intracellular [Ca^2+^] and thus contribute to elevated levels in the cytosol (Figure 3 and Figure 8b). It is also possible that the Ca^2+^ ejection and buffering mechanisms are weakened in neurons after STIM silencing. Downregulating STIM2 [22,66] or STIM1 [67,68] can induce Ca^2+^ overload and destabilization of hippocampal mushroom spines in AD or aged neurons. Downregulating STIM2 proteins was also observed in cells from AD patients [69]. In turn, the overexpression of STIMs leads to recovery of normal spine morphology and reduced intracellular Ca^2+^ overload in AD neurons [22,66,68,70]. These studies are consistent with our observations that overexpressing STIM1 or STIM2 in cortical neurons and in neurons from STIM1-overexpressing mice was sufficient to reduce cytosolic Ca^2+^ levels induced by NMDA treatment (Figure 4 and Figure 8c). We suspect that overexpressing STIMs partially prevented NMDA-mediated Ca^2+^ responses, which suppressed intracellular Ca^2+^ overload.

## 5. Conclusions

In conclusion, our results demonstrate that NMDAR, in addition to Orai, TRPC, VGCC, and AMPAR, comprise putative Ca^2+^ sources for nSOCE [21,31]. Moreover, our findings presented here reveal a previously unidentified function of STIMs in neuronal signaling by interacting with NMDARs. Our data suggest that STIM1 and STIM2 are negative regulators of NMDA-evoked intracellular Ca^2+^ elevations in cortical neurons, as is known for VGCC activity [27,28]. We propose that upregulating STIMs can protect against NMDA-induced dysfunctions of Ca^2+^ homeostasis. Thus, our results advance our knowledge on the pathophysiological mechanisms of neurodegenerative diseases. 

## Figures and Tables

**Table 1:** Experimental design.

Type of Experimental Material	Type of Experimental Method	Experiment Variants
HEK 293T/17 cells	Lentivirus production	-
HeLa cells	[Ca^2+^]_i_ measurements	SOCE +/− memantine (MM)
Brain slices from P28 mouse (WT/Tg(STIM1)Ibd)	Electrophysiology	NMDAR activation
Primary neuronal cultures from E19 mice (WT/Tg(STIM1)Ibd) brains	[Ca^2+^]_i_ measurements (DIV17)	NMDA + glycine
Primary neuronal cultures from E19 Wistar rat brains	Co-IP (DIV15)	+/− thapsigargin
	IF, PLA (DIV17)	
	[Ca^2+^]_i_ measurements (DIV17)	SOCE +/− TTX, +/− D-AP5/MM
	[Ca^2+^]_i_ measurements (DIV16)	NMDA + glycine +/− D-AP5/MM/SKF96365
	shRNA virus transduction (DIV4) and [Ca^2+^]_i_ measurements (DIV16)	NMDA + glycine
	YFP transfection (DIV15) and [Ca^2+^]_i_ measurements (DIV16)	NMDA + glycine

NMDAR: *N*-methyl-d-aspartate receptor; STIM1: Stromal interacting molecule 1; SOCE: store-operated Ca^2+^ entry.

## Supplementary Materials

The following are available online at https://www.mdpi.com/2073-4409/9/1/160/s1, Figure S1: In vitro quantification of NMDA-induced currents with STIM overexpression, Table S1: Interaction between STIMs and NR2 subunits of the NMDA receptor.
