# Supplementary material for: STIM Protein-NMDA2 Receptor Interaction Decreases NMDA-Dependent Calcium Levels in Cortical Neurons
Source: Cells. 2020 Jan 9;9(1):160. doi: 10.3390/cells9010160 (PMC7017226; doi:10.3390/cells9010160)
Supplement: Supplementary file 1 [file cells-09-00160-s001.zip › cells-682504-supplementary.pdf]

Electronic Supplementary Material

STIM protein-NMDA2 receptor interaction decreases NMDA-dependent calcium levels in cortical neurons

Joanna Gruszczynska-Biegala[1,2,*], Klaudia Strucinska[1], Filip Maciag[1], Lukasz Majewski[1], Maria Sladowska[1,a], Jacek Kuznicki[1]

[1]International Institute of Molecular and Cell Biology, Laboratory of Neurodegeneration, Warsaw, Poland

[2]Mossakowski Medical Research Centre Polish Academy of Sciences, Molecular Biology Unit, Warsaw, Poland

[a] present address: The Centre of New Technologies, Laboratory of Mitochondrial Biogenesis, Warsaw, Poland

* **corresponding author**: jgruszczynska@imdik.pan.pl Orcid: 0000-0003-3541-2550

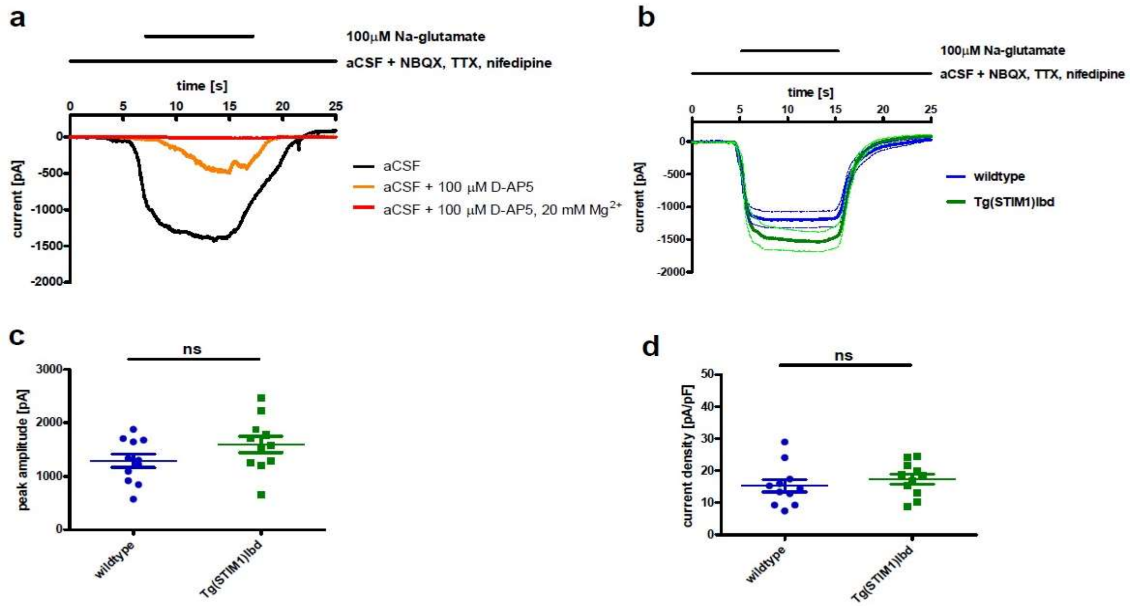

Figure S1. *In vitro* quantification of NMDA-induced currents with STIM overexpression. Electrophysiological recordings of NMDA-induced currents recorded from layer V pyramidal neurons from acute brain slices isolated from wild type and *Tg*(Stim1)Ibd mice. $n_{wild\text{-}type} = n_{Tg(Stim1)Ibd} = 11$. (a) Inhibiting evoked currents by D-AP5 and Mg^{2+} ions (traces from single experiment, all stimulations performed on the same cell). (b) Averaged traces of responses obtained from neurons of the two genetic variants; dotted lines represent standard deviation of the mean. (c) Comparison of peak amplitude of NMDAR-mediated currents; ns – not significant ($p < 0.05$). (d) Comparison of current density of NMDAR-mediated currents ($p < 0.05$).

Table S1. Interaction between STIMs and NR2 subunits of the NMDA receptor. Arrows indicate statistically significant change (increase or decrease), NC - no statistically significant change.

**Interacting proteins**	**PLA** TG/EGTA *vs* Ca^{2+}	**Immunofluorescence** TG/EGTA *vs* Ca^{2+}	**Co-IP** TG/EGTA *vs* Ca^{2+}
STIM1 with NR2A	NC	⇧	⇧ or NC
STIM1 with NR2B	⇩	⇩	⇩
STIM2 with NR2A	⇩	⇩	⇩
STIM2 with NR2B	⇧	NC	NC
